# Comparative Chloroplast Genomics of *Cousinia* (Asteraceae) With Nine Newly Sequenced Endemic Species From Central Asia

**DOI:** 10.1002/ece3.73589

**Published:** 2026-04-29

**Authors:** Bobur Karimov, Diyorjon Hamrayev, Husniddin Esanov, Alijon Eshonkulov, Oybek Omonov, Nodira Boboyeva, Damira Karimova, Abdullajon Umedov, Temur Asatulloev, Ziyoviddin Yusupov, Komiljon Sh. Tojibaev

**Affiliations:** ^1^ Institute of Botany Academy of Sciences of Uzbekistan Tashkent Uzbekistan; ^2^ Bukhara State University Bukhara Uzbekistan; ^3^ Bukhara State Medical Institute Bukhara Uzbekistan; ^4^ Karshi State University Karshi Uzbekistan; ^5^ Turan University Karshi Uzbekistan; ^6^ Termez State University Termez Uzbekistan; ^7^ Jizzakh State Pedagogical University Jizzakh Uzbekistan

**Keywords:** anther appendage morphology, codon usage bias, nucleotide diversity, Pamir–Alay, phylogenetic incongruence, plastid genome evolution, simple sequence repeats

## Abstract

*Cousinia* (Asteraceae: Cardueae) represents one of the most species‐rich genera within the Irano–Turanian floristic region, yet interspecific relationships remain incompletely resolved. Here, nine chloroplast genomes from species endemic to the Pamir–Alay mountain system were newly sequenced and analyzed in combination with previously published complete chloroplast (cp) genome sequences and nuclear ribosomal DNA (nrDNA) data. The assembled cp genomes were highly uniform in size (approximately 152 kb), displayed the quadripartite organization of angiosperm chloroplasts, and possessed a GC content of 37.7%. Gene composition was largely conserved, with 131 annotated genes identified in most taxa. Examination of synonymous codon usage across 16 cp genomes revealed a consistent bias toward A/T‐ending codons. Sliding‐window analysis demonstrated generally low nucleotide diversity (Pi = 0–0.00918), although several divergence hotspots were detected, primarily within the large and small single‐copy regions. Eleven categories of simple sequence repeats were identified, with A/T‐rich mononucleotide motifs predominating. Phylogenetic reconstruction based on cp genomes data did not consistently recover morphologically defined sections as monophyletic, whereas the nrDNA dataset provided improved resolution of sectional delimitations. Comparative analysis of anther appendage morphology recognized nine structural groups and showed partial congruence with molecular evidence. Together, these findings highlight incongruence between chloroplast and nuclear signals and indicate that certain infrageneric classifications within *Cousinia* warrant re‐evaluation.

## Graphical abstract

In this study, nine complete chloroplast genomes of Cousinia species endemic to the Pamir–Alay mountain system were sequenced and analyzed together with previously published plastomes and nuclear ribosomal ITS data.

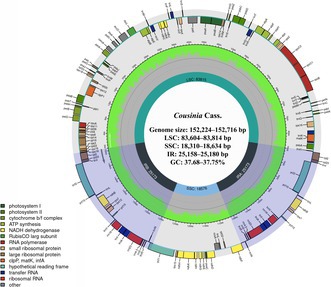

## 1 Introduction

Mountain systems of the Irano–Turanian floristic region represent one of the most important global centers of plant diversification (Manafzadeh et al. 2017), particularly for xerophytic and montane lineages within Asteraceae. Among these, the genus *Cousinia* Cass. stands out as an exceptional example of rapid radiation and taxonomic complexity. With approximately 673 currently accepted species (POWO 2026), *Cousinia* constitutes one of the largest genera in the tribe Cardueae and is predominantly distributed across Central and Western Asia. The highest concentration of species richness occurs in the Pamir–Alay, Tianshan, and Iranian Plateau mountain systems, areas widely recognized for their high endemism and ecological heterogeneity (Knapp 1987; Tscherneva 1993). Paleoecological evidence indicates that *Cousinia* persisted throughout Quaternary glacial periods and was likely more widespread during these intervals, suggesting a high degree of resilience of Irano–Turanian floristic elements to past climatic fluctuations (Djamali et al. 2012).

Understanding evolutionary relationships within such species‐rich, geographically structured lineages requires robust genomic data capable of resolving fine‐scale patterns of divergence. Complete chloroplast (cp) genome sequencing has emerged as a powerful approach for investigating genome evolution and resolving complex phylogenetic patterns. Cp genome‐scale data provide comprehensive information on genome organization, gene content, structural variation, codon usage bias, simple sequence repeats (SSRs), and nucleotide divergence patterns. In Asteraceae, recent cp genome studies have significantly enhanced phylogenetic resolution and contributed to improved understanding of evolutionary relationships within morphologically diverse genera (Yang et al. 2023; Zhong et al. 2023; Mahai et al. 2024; Nyamgerel et al. 2024; Karimov, Yuldashev, et al. 2025; Abdullah et al. 2025, 2026; Xing et al. 2025).

In *Cousinia*, previous cp genome and integrative studies remain limited in both taxonomic breadth and geographic representation, particularly with respect to the Pamir–Alay region, a major center of diversification and endemism. For instance, Karimov, Tojibaev, et al. (2025) analyzed cp genomes of six species, together with one previously available cp genome from NCBI, representing three sections (*Alpinae*, *Homalochaete*, and *Dichotomae*), and examined anther appendage morphology in 20 species from the same groups. Although this study employed an integrative framework combining cp genome, nrDNA, and morphological data, its restricted sampling does not adequately reflect the extensive sectional diversity and high endemism of Pamir–Alay *Cousinia*. Consequently, cp genome variation, genomic differentiation, and phylogenetic relationships within this key diversification center remain insufficiently resolved. This limitation prompts the following research question: Does expanded taxonomic and sectional sampling of Pamir–Alay endemic species, combined with cp genome, nrDNA, and morphological data, improve phylogenetic resolution and clarify the congruence between molecular and morphology‐based classifications in *Cousinia*?

To address this question, we expand both genomic and morphological datasets by incorporating nine newly sequenced cp genomes from Pamir–Alay endemic species representing a broader range of sections (*Regelianae*, *Coronophora*, *Acanthotoma*, *Olgaeanthe*, *Tianschanicae*, and *Helianthae*), together with additional representatives of previously studied groups. Morphological sampling is further extended through the examination of anther appendage characters in more than 40 species. These data are analyzed in combination with previously available cp genome and nrDNA sequences within a unified framework to: (1) characterize cp genome structure and codon usage patterns; (2) identify highly variable regions and SSR loci; (3) assess interspecific genetic divergence; and (4) reconstruct phylogenetic relationships and evaluate their congruence with traditional morphology‐based classifications.

## 2 Material and Methods

### 2.1 Plant Material

**FIGURE 1:**
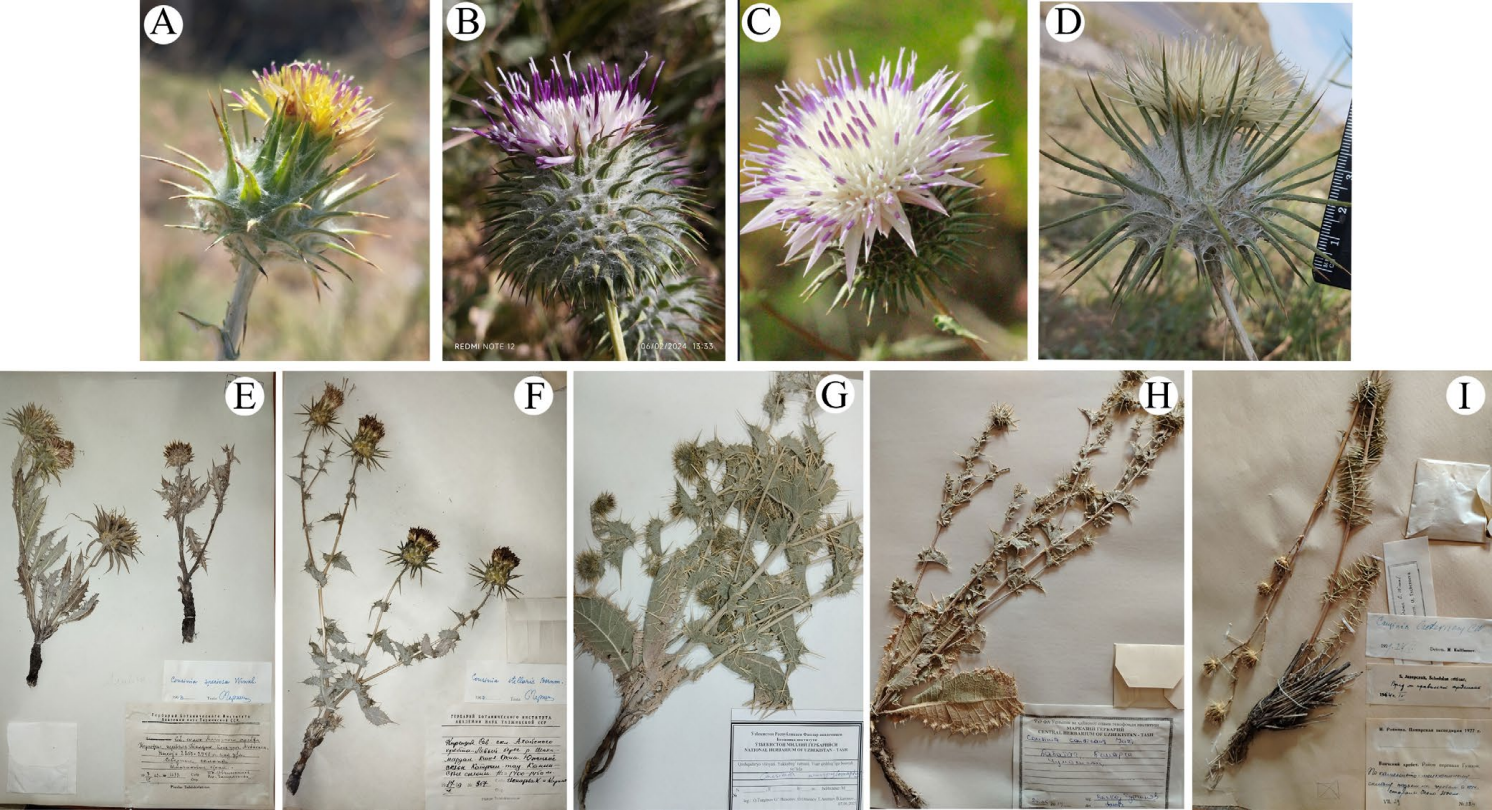
*Cousinia* species sampled for DNA extraction and sequencing in this study. (A) *Cousinia botschantzevii*, (B) *Cousinia integrifolia*, (C) *Cousinia coronata*, (D) *Cousinia spryginii*, (E) *Cousinia speciosa*, (F) *Cousinia stellaris*, (G) *Cousinia campylaraphis*, (H) *Cousinia candicans*, (I) *Cousinia laetevirens*.

Nine species representing eight infrageneric sections of *Cousinia* from the Pamir–Alay region were selected to reflect major evolutionary lineages (Table 1 and Figure 1). Fresh leaf material was collected from natural populations in Uzbekistan during 2023–2025 and dried in silica gel. When field sampling was not feasible, DNA was extracted from herbarium specimens deposited in the National Herbarium of Uzbekistan (TASH) and the Herbarium of the Institute of Botany, Plant Physiology and Genetics of the National Academy of Sciences of Tajikistan (TAD), with prior curator authorization.

**TABLE 1:** Species, sectional placement, distribution, voucher data, and NCBI accession numbers for *Cousinia* cp genome samples sequenced in this study.

Species	Species Section and General distribution	Voucher Information	NCBI accession no. cp and nrDNA
*C. botschantzevii* Juz. ex Tscherneva	Sect. *Regelianae* (Juz.) Tschern.; endemic to the Pamir–Alay (Nuratau Mountains)	Uzbekistan, Navoiy Region, Nuratau Mountains. 11 May 2025. Leg. Karimov B. Observation link: https://www.inaturalist.org/observations/280210636	PX230056 PX632335
*C. campylaraphis* Tschern.	Sect. *Homalochaete* C. Winkl.; endemic to the Pamir–Alay (Southwestern Hissar)	Uzbekistan, Kashkadarya Region, road to Vuar village. 7 June 2023. Leg. Turginov et al. (TASH)	PX230055 PX632341
*C. candicans* Juz.	Sect. *Homalochaete* C. Winkl.; endemic to the Pamir–Alay (Babatag Range)	Uzbekistan, Surkhondarya Region, Babatag Mountains. 25 May 2019. Leg. Beshko et al. (TASH)	PX230051 PX632343
*C. coronata* Franch.	Sect. *Coronophora* (Juz.) Rech. f.; endemic to the Pamir–Alay	Uzbekistan, Surkhondarya Region, Topalang River basin, vicinity of Bakhcha village. 14 May 2023. Leg. Turdiboev et al. (TASH)	PX230054 PX632330
*C. laetevirens* C. Winkl.	Sect. *Acanthotoma* Juz.; endemic to the Pamir–Alay	Tajikistan, Vanch Range, southern approach to the Gushkhon Pass, stony light‐loam slope, ca. 3500 m. July 1929. No. 1121 (TASH)	PX230053 PX632334
*C. integrifolia* Franch.	Sect. *Olgaeanthe* Tschern.; endemic to the Pamir–Alay	Uzbekistan, Samarkand Region, vicinity of Aman‐Kutan Pass. 2 Jun 2024. Leg. Karimov B. Observation link: https://www.inaturalist.org/observations/219948664	PX230050 PX632340
*C. speciosa* C. Winkl.	Sect. *Tianschanicae* Sennikov; endemic to the Pamir–Alay (Alay and Zaalayskiy Ranges)	Kyrgyzstan, Northern slope of the Alay Range, foot of the Taldyk Pass, near Ak‐Bashi pasture, 2850–2940 m. 7 September 1952. No. 1173, Leg. Ovchinnikov et al. (TAD)	PX230049 PX632331
*C. stellaris* Bornm.	Sect. *Alpinae* Bunge; endemic to the Pamir–Alay (Alay Range)	Kyrgyzstan, Northern slope of the Alay Range, left bank of the Shakhimardan River, Okhna village, rocky slopes, 1400–1450 m. 17 June 1959. No. 357, Leg. Ismatova et al. (TAD)	PX230048 PX632332
*C. spryginii* Kult.	Sect. *Helianthae* Bge.; endemic to the Pamir–Alay (Southwestern Hissar)	Uzbekistan, Kashkadarya Region, Dehkanabad District, near roadside. 24 May 2024. Leg. Karimov B. Observation link: https://www.inaturalist.org/observations/217885461	PX230047 PX632333

### 2.2 DNA Extraction and Sequencing

Genomic DNA was isolated from leaf tissue using the Tiangen DP305 Plant Genomic DNA Kit (Beijing, China). Libraries were prepared with the NEBNext Ultra DNA Library Prep Kit for Illumina (Cat. E7370L; NEB, USA) following the manufacturer's protocol, with index codes added during preparation. DNA was sonicated to ~350 bp, and fragments were end‐repaired, A‐tailed, and ligated to Illumina adapters, followed by PCR amplification. PCR products were purified using AMPure XP beads (Beverly, USA). Library quality was assessed on an Agilent 5400 system, and concentrations were quantified by qPCR (1.5 nM). Qualified libraries were pooled and sequenced on Illumina platforms using the PE150 strategy at Novogene (Beijing, China).

### 2.3 Genome Assembly and Annotation

High‐quality paired‐end reads were assembled de novo with NOVOPlasty v4.3.5 (Dierckxsens et al. 2017), using the cp genome of *Cousinia rotundifolia* (PQ240609) as a seed reference. This procedure produced a cp genome for each sampled species. To validate assemblies, filtered reads were aligned against their corresponding cp genomes using BWA‐MEM v0.7.17 (Li 2013). Resulting alignments were processed, sorted, and indexed with SAMtools v1.19.2 (Li et al. 2009), and sequencing depth across sites was calculated within the same package. Genome annotation was conducted through GeSeq (Tillich et al. 2017) with reference guidance from *C. rotundifolia* . Gene boundaries were subsequently verified and, where necessary, adjusted in Geneious v9.0.2 (Kearse et al. 2012), with particular attention to coding region start/stop positions and intron–exon structures. The physical representation of each cp genome was visualized using Chloroplot (Zheng et al. 2020). For the assembly of nrDNA, the sequence of *C. proxima* (PX632342; 5821 bp) was employed as the seed reference.

### 2.4 Comparative Genomic Analyses

Codon usage patterns and potential biases were investigated across 16 complete *Cousinia* cp genomes. Protein‐coding regions were retrieved using Biopython v1.83 (Cock et al. 2009). To avoid redundancy, one copy of genes duplicated within the inverted repeat regions was retained. All sequences were verified for correct open reading frames and screened to exclude internal stop codons. Codon counts were derived from concatenated coding regions for each species. Termination codons (TAA, TAG, TGA), amino acids encoded by a single codon (Met and Trp), and the infrequently used plastid arginine codons (AGA, AGG) were omitted from subsequent calculations. Relative Synonymous Codon Usage (RSCU) indices were then determined to assess synonymous codon preference.

Levels of nucleotide diversity (Pi) across cp genomes were evaluated in DnaSP v6.11 (Rozas et al. 2017) using a sliding window approach with an 800 bp window and 200 bp increment. Microsatellite loci were detected using MISA (Beier et al. 2017), applying minimum repeat thresholds of 10 for mononucleotides, 5 for dinucleotides, 4 for trinucleotides, and 3 for tetra‐, penta‐, and hexanucleotide motifs.

For comparative analyses, SSR frequencies were compiled into a species‐by‐motif matrix. Compositional differences among taxa were quantified using Bray–Curtis distances (Bray and Curtis 1957), followed by hierarchical clustering under the Ward D2 criterion (Ward 1963; Murtagh and Legendre 2014) to generate a phenetic dendrogram based on microsatellite profiles.

Additionally, comparative analyses were conducted within the Chloroplast Genome Analysis Suite (CGAS) framework (Abdullah, Yan, and Tian 2026), which integrates multiple bioinformatics tools, including GetOrganelle (Jin et al. 2020), PGA (Qu et al. 2019), MAFFT (Katoh and Standley 2013), IQ‐TREE (Nguyen et al. 2015), Biopython (Cock et al. 2009), and R (R Core Team 2023), ensuring a standardized and reproducible workflow.

### 2.5 Phylogenetic Analysis

Phylogenetic reconstruction was based on two independent molecular datasets: cp genome sequences and nrDNA regions. The cp genome dataset included 19 taxa, comprising nine newly sequenced species and 10 accessions retrieved from GenBank. *Arctium lappa* , *Jurinea auriculata*, and *Dolomiaea wardii* were designated as outgroups. The nrDNA matrix consisted of 18 taxa (nine newly generated and nine obtained from GenBank), with *Carduus acanthoides* , *Erigeron philadelphicus* , and *Atractylodes lancea* selected for rooting purposes. The cp genome alignment covered 154,308 bp, including 1995 variable sites and 764 parsimony‐informative positions. The nrDNA alignment spanned 5844 bp, of which 195 sites were variable and 88 parsimony‐informative. Alignments were manually checked, and insertion–deletion events were treated as missing data. Each dataset was analyzed independently. Maximum Likelihood analyses were implemented in RAxML v8.2.12 (Stamatakis 2014). The cp genome dataset was evaluated under the GTR + G substitution model with 1000 bootstrap replicates. For the nrDNA matrix, jModelTest v2.1.10 (AICc criterion) selected GTR + I + G as the optimal model, which was subsequently applied in RAxML with 1000 bootstrap replicates. Bayesian inference was performed using MrBayes v3.2.7 (Ronquist et al. 2012), running two independent MCMC analyses for 10 million generations with sampling every 1000 generations. Convergence was confirmed when the average standard deviation of split frequencies fell below 0.01, and the initial 25% of sampled trees were discarded as burn‐in. Final phylogenetic trees were visualized and annotated in iTOL v6 (Letunic and Bork 2021).

## 3 Results

### 3.1 Sequencing and Assembly

Sequencing output and read‐mapping statistics were evaluated to assess data quality and ensure the reliability of cp genome assembly. The total number of reads per species ranged from 16,964,951 in *C. coronata* to 680,809,153 in *C. campylaraphis* (Table 2). The proportion of mapped reads ranged from 1.23% (*C. botschantzevii*) to 9.95% ( *C. stellaris* ). Properly paired reads showed a similar range, from 1.10% in *C. botschantzevii* to 9.74% in *C. stellaris* . Mean sequencing depth differed substantially among species, with the lowest depth observed in *C. coronata* (1084.56×) and the highest in *C. campylaraphis* (30,784.2×). High coverage was also obtained for *C. candicans* (19,146.3×) and *C. stellaris* (13,586×), whereas moderate coverage levels were recorded for *C. integrifolia* (7260.84×), *C. spryginii* (7260.88×), *C. speciosa* (2961.02×), *C. laetevirens* (2341.92×), and *C. botschantzevii* (1668.21×). Overall, all species achieved sufficient sequencing depth to support reliable cp genome assembly.

**TABLE 2:** Summary of read mapping statistics for cp genome sequencing of *Cousinia* species.

Species	Total reads	Mapped (%)	Properly paired (%)	Mean depth
*C. integrifolia*	148,619,123	5.20	4.98	7260.84
*C. spryginii*	157,978,956	4.83	4.64	7260.88
*C. botschantzevii*	153,580,807	1.23	1.10	1668.21
*C. laetevirens*	152,724,222	1.77	1.63	2341.92
*C. stellaris*	142,085,434	9.95	9.74	13,586
*C. speciosa*	146,445,132	2.30	2.18	2961.02
*C. candicans*	628,412,024	3.26	3.09	19,146.3
*C. campylaraphis*	680,809,153	4.70	4.53	30,784.2
*C. coronata*	16,964,951	6.72	6.41	1084.56

### 3.2 Chloroplast Genome Features

**FIGURE 2:**
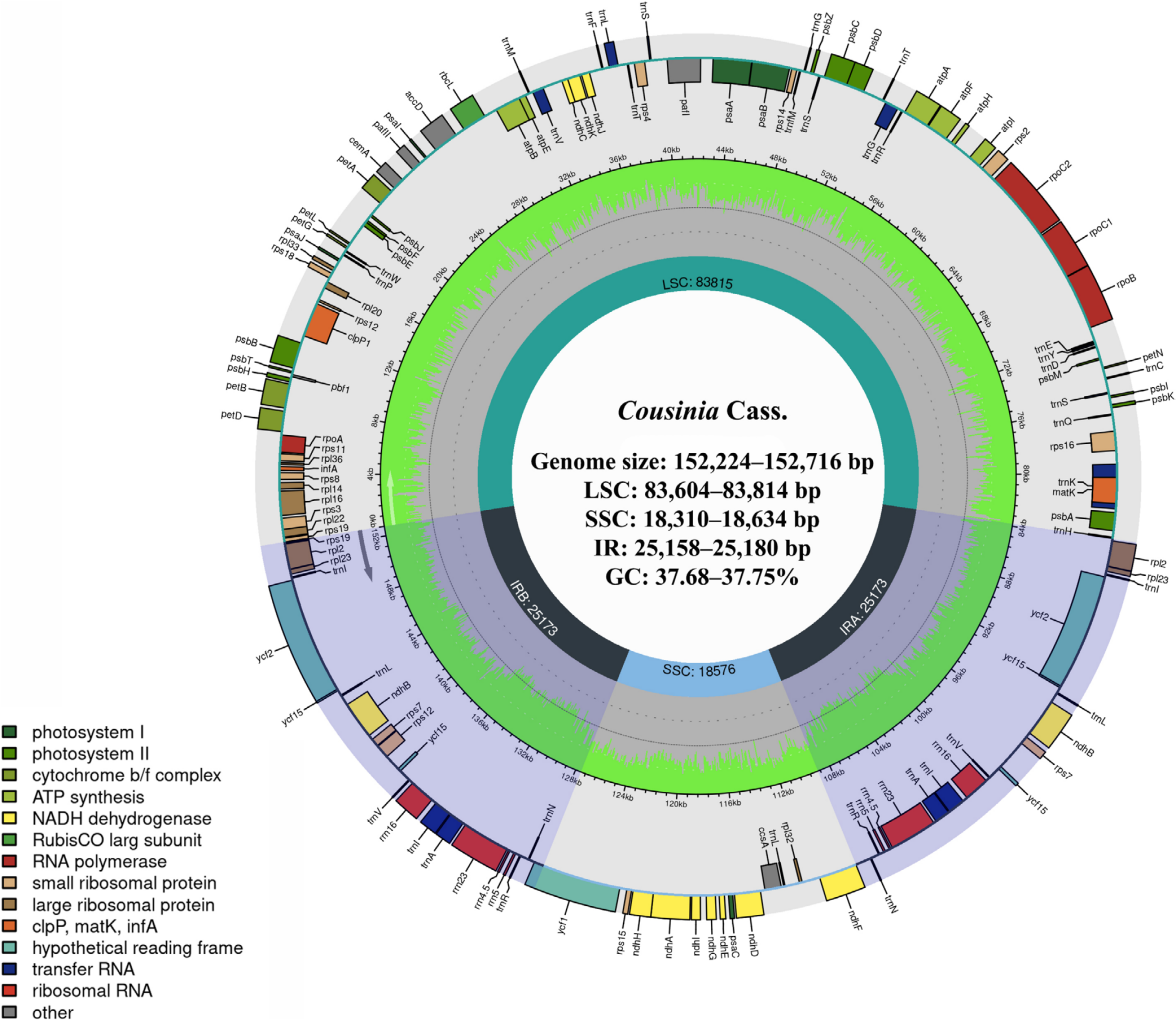
Circular map of the chloroplast genome of *Cousinia spryginii* was drawn using the Chloroplot to show the genes present in each region (LSC, SSC, and IRs). The transcription directions for the inner and outer genes are clockwise and anticlockwise, respectively, and each functional group of genes is distinctively color‐marked. In the inner circle, the green shades represent the GC content, and the lighter gray shades signify the AT content.

The cp genomes of the examined *Cousinia* species exhibited a high degree of structural conservation in both genome size and GC content (Table 3). Genome lengths ranged narrowly from 152,224 bp in *C. thomsonii* to 152,716 bp in *C. spryginii*, indicating minimal size variation across the dataset. All cp genomes displayed the typical quadripartite structure (Figure 2), consisting of a large single‐copy (LSC) region (83,604–83,814 bp), a small single‐copy (SSC) region (18,310–18,634 bp), and a pair of inverted repeat (IR) regions (25,158–25,180 bp). The overall GC content was highly conserved, varying only slightly between 37.68% ( *C. rhodantha* , *C. rotundifolia* , *C. stellaris* , *C. speciosa* , *C. integrifolia* , and *C. botschantzevii*) and 37.75% ( *C. thomsonii* ). Region‐specific GC content followed a consistent pattern across all species, with the IR regions exhibiting the highest GC content (43.09%–43.12%), followed by the LSC region (35.81%–35.88%), and the SSC region showing the lowest values (31.33%–31.55%). At the gene level, GC content was also relatively stable among species. The GC content of tRNA genes ranged from 52.72% to 53.06%, while rRNA genes showed consistently high GC content (54.58%–54.60%). Protein‐coding sequences (CDS) exhibited lower GC values, ranging from 37.95% to 38.01%. These results indicate strong conservation of nucleotide composition across the cp genomes of *Cousinia* species.

**TABLE 3:** Summary of chloroplast genome size, structural regions, and GC content across *Cousinia* species.

Species	Genome length (bp)				GC (%)				GC (%)			Accession number
	Complete	LSC	SSC	IR	Complete	LSC	SSC	IR	tRNA	rRNA	CDS	
*C. thomsonii*	152,224	83,605	18,310	25,165	37.75	35.87	31.55	43.12	53.02	54.60	37.99	PP525141
*C. rhodantha*	152,581	83,639	18,604	25,179	37.68	35.82	31.36	43.10	52.91	54.58	37.96	PQ152229
*C. proxima*	152,598	83,724	18,548	25,171	37.72	35.85	31.49	43.11	53.02	54.58	38.00	PQ240605
*C. orthacantha*	152,533	83,687	18,529	25,169	37.72	35.84	31.49	43.11	53.02	54.58	38.00	PQ240607
*C. rotundifolia*	152,538	83,607	18,592	25,180	37.68	35.81	31.35	43.09	52.91	54.58	37.95	PQ240609
*C. pseudodshizakensis*	152,532	83,604	18,590	25,179	37.69	35.82	31.38	43.10	52.91	54.58	37.96	PQ240610
*C. subcandicans*	152,576	83,662	18,583	25,176	37.73	35.88	31.46	43.11	53.02	54.58	38.01	PQ364117
*C. spryginii*	152,716	83,814	18,575	25,174	37.69	35.83	31.37	43.10	52.72	54.58	37.97	PX230047
*C. stellaris*	152,556	83,653	18,564	25,180	37.68	35.82	31.33	43.10	52.91	54.58	37.95	PX230048
*C. speciosa*	152,593	83,664	18,590	25,180	37.68	35.81	31.37	43.10	52.91	54.58	37.96	PX230049
*C. integrifolia*	152,539	83,608	18,592	25,180	37.68	35.81	31.35	43.09	52.91	54.58	37.95	PX230050
*C. candicans*	152,535	83,657	18,559	25,158	37.72	35.86	31.49	43.11	53.02	54.58	38.01	PX230051
*C. laetevirens*	152,613	83,671	18,631	25,166	37.72	35.86	31.46	43.12	53.02	54.58	38.01	PX230053
*C. coronata*	152,563	83,660	18,570	25,177	37.73	35.86	31.51	43.11	53.06	54.58	38.01	PX230054
*C. campylaraphis*	152,646	83,683	18,634	25,175	37.72	35.86	31.47	43.12	53.02	54.58	38.01	PX230055
*C. botschantzevii*	152,681	83,812	18,544	25,173	37.68	35.82	31.37	43.09	52.72	54.58	37.96	PX230056

Abbreviations: CDS, protein‐coding gene; Complete, complete chloroplast genome; GC, guanine‐cytosine content; IR, inverted repeat region; LSC, large single‐copy region; rRNA, ribosomal RNA; SSC, small single‐copy region; tRNA, transfer RNA.

Each of the nine cp genomes encoded a total of 131 genes (113 unique), comprising 87 protein‐coding genes (PCGs), 36 transfer RNA (tRNA) genes, and eight ribosomal RNA (rRNA) genes, with no detectable variation in overall gene content among species. Eighteen loci were duplicated as a consequence of their position within the inverted repeat (IR) regions. These included seven tRNA genes (*trnA*‐UGC, *trnI*‐CAU, *trnI*‐GAU, *trnL*‐CAA, *trnN*‐GUU, *trnR*‐ACG, *trnV*‐GAC), four rRNA genes (*rrn16*, *rrn23*, *rrn4.5*, *rrn5*), and seven protein‐coding genes (*ndhB*, *rpl2*, *rpl23*, *rps12*, *ycf2*, *ycf15*, *rps7*), consistent with the conserved organization of angiosperm cp genomes.

A total of 16 genes contained introns, of which 11 were protein‐coding (*atpF*, *rpoC1*, *rpl2*, *ndhB*, *ndhA*, *petB*, *petD*, *rps16*, *rps12*, *ycf3*, *clpP*) and five were tRNA genes (*trnA*‐UGC, *trnI*‐GAU, *trnK*‐UUU, *trnL*‐UAA, *trnV*‐UAC). Among these, *ycf3* and *clpP* harbored two introns each, whereas the remaining intron‐bearing genes contained a single intron.

### 3.3 Codon Usage

**FIGURE 3:**
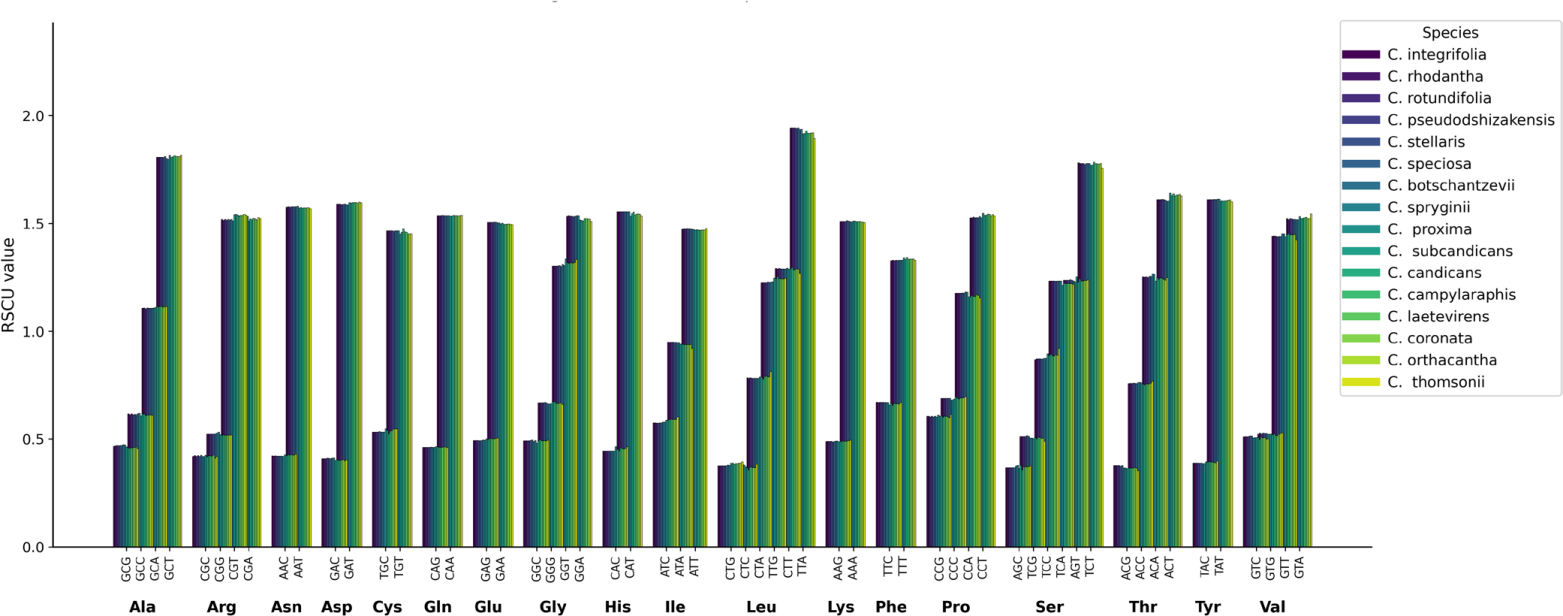
Relative synonymous codon usage (RSCU) patterns across the chloroplast genomes of 16 *Cousinia* species.

Analysis of RSCU across the cp genomes of 16 *Cousinia* species revealed highly conserved codon usage patterns, with only minor interspecific variation (Figure 3 and File S1). Across all species, codon usage showed a strong bias toward A/T‐ending codons, whereas G/C‐ending codons were consistently underrepresented. This AT bias was evident for nearly all amino acids and is consistent with the AT‐rich nucleotide composition typical of cp genomes. Amino acids encoded by two synonymous codons displayed pronounced directional bias. For example, AAT (Asn; RSCU ≈ 1.57) greatly exceeded AAC (≈ 0.42), and GAT (Asp; ≈ 1.59) was strongly favored over GAC (≈ 0.40). Similar patterns were observed for Cys (TGT > TGC), Gln (CAA > CAG), Glu (GAA > GAG), His (CAT > CAC), Phe (TTT > TTC), Tyr (TAT > TAC), and Lys (AAA ≈ 1.51 vs. AAG ≈ 0.49). Amino acids encoded by four or six synonymous codons also exhibited clear codon usage asymmetry. For alanine, GCT was the most preferred codon (RSCU ≈ 1.80), whereas GCC and GCG were underused (≈0.45–0.62). Glycine showed moderate bias, with GGT and GGA favored (≈1.33–1.53) and GGC underrepresented (≈0.49). Leucine displayed one of the strongest biases, with TTA (≈1.90) and TTG (≈1.22) being highly preferred, while CTG and CTC showed consistently low RSCU values (≈0.37–0.38). Similar A/T‐ending codon preferences were observed for serine (TCT ≈ 1.77), proline (CCT ≈ 1.53), threonine (ACT ≈ 1.61), and valine (GTA and GTT ≈ 1.44–1.54).

### 3.4 Nucleotide Diversity

**FIGURE 4:**
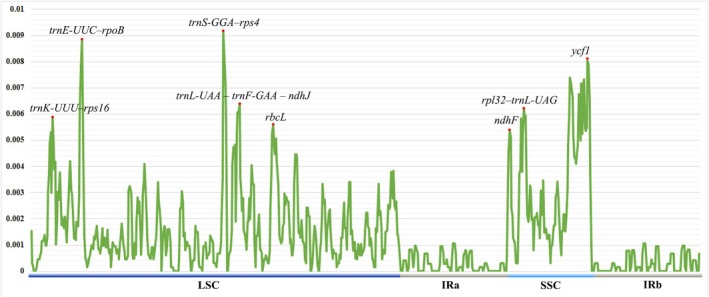
Variation in nucleotide diversity (π) across LSC, IR, and SSC regions of *Cousinia* chloroplast genomes.

Sliding‐window analysis cp genomes with an 800 bp window and 200 bp increment revealed an uneven distribution of nucleotide diversity across the cp genomes of the 16 *Cousinia* species examined. Overall Pi values were low (0–0.00918), reflecting the highly conserved nature of cp genomes (Figure 4). Nevertheless, several distinctly variable regions were identified. Most variable nucleotides were concentrated in the LSC and SSC regions, whereas the IR regions showed the highest degree of stability and exhibited the lowest levels of diversity.

Within the LSC region, five major peaks of variation were detected: *trnK‐UUU–rps16*, *trnE‐UUC–rpoB*, *trnS‐GGA–rps4*, *trnL‐UAA–trnF‐GAA–ndhJ*, and a moderate variability peak within the *rbcL* gene. In the SSC region, three pronounced hotspots were identified, corresponding to the *ndhF* gene, the *rpl32–trnL‐UAG* intergenic spacer, and the *ycf1* gene.

Nucleotide diversity was further assessed across coding and noncoding regions of the cp genomes (Figure S1). Overall, sequence variation was low, indicating a high level of conservation among the analyzed regions. Most protein‐coding and tRNA genes, including *trnH‐GUG*, *psbA*, *trnK‐UUU*, *psbK*, and *psbI*, were completely conserved (π = 0). Moderate variation was detected in *matK* (π = 0.00077), *rps16* (π = 0.00209), and *rbcL* (π = 0.00565), while the highest nucleotide diversity among coding regions occurred in *trnF‐GAA* (π = 0.01416), *trnP‐UGG* (π = 0.00676), and *ycf1* (π = 0.00605). Ribosomal RNA genes (*16S*, *23S*, *4.5S*, and *5S rRNA*) were fully conserved. Noncoding regions, including intergenic spacers and introns, displayed higher levels of variation, with notable hotspots at *trnL‐UAG–ccsA* (π = 0.01444), *rps11–rpl36* (π = 0.01135), *psbC–trnS‐UGA* (π = 0.00912), *ycf3–trnS‐GGA* (π = 0.00808), and *trnE‐UUC–rpoB* (π = 0.00843). Several additional regions, such as *trnT‐UGU–trnL‐UAA* (π = 0.00627), *psaC–ndhE* (π = 0.00677), and *rps15–ycf1* (π = 0.00638), also exhibited elevated nucleotide diversity, highlighting the variable nature of non‐coding sequences relative to coding regions.

### 3.5 SSR Markers

**FIGURE 5:**
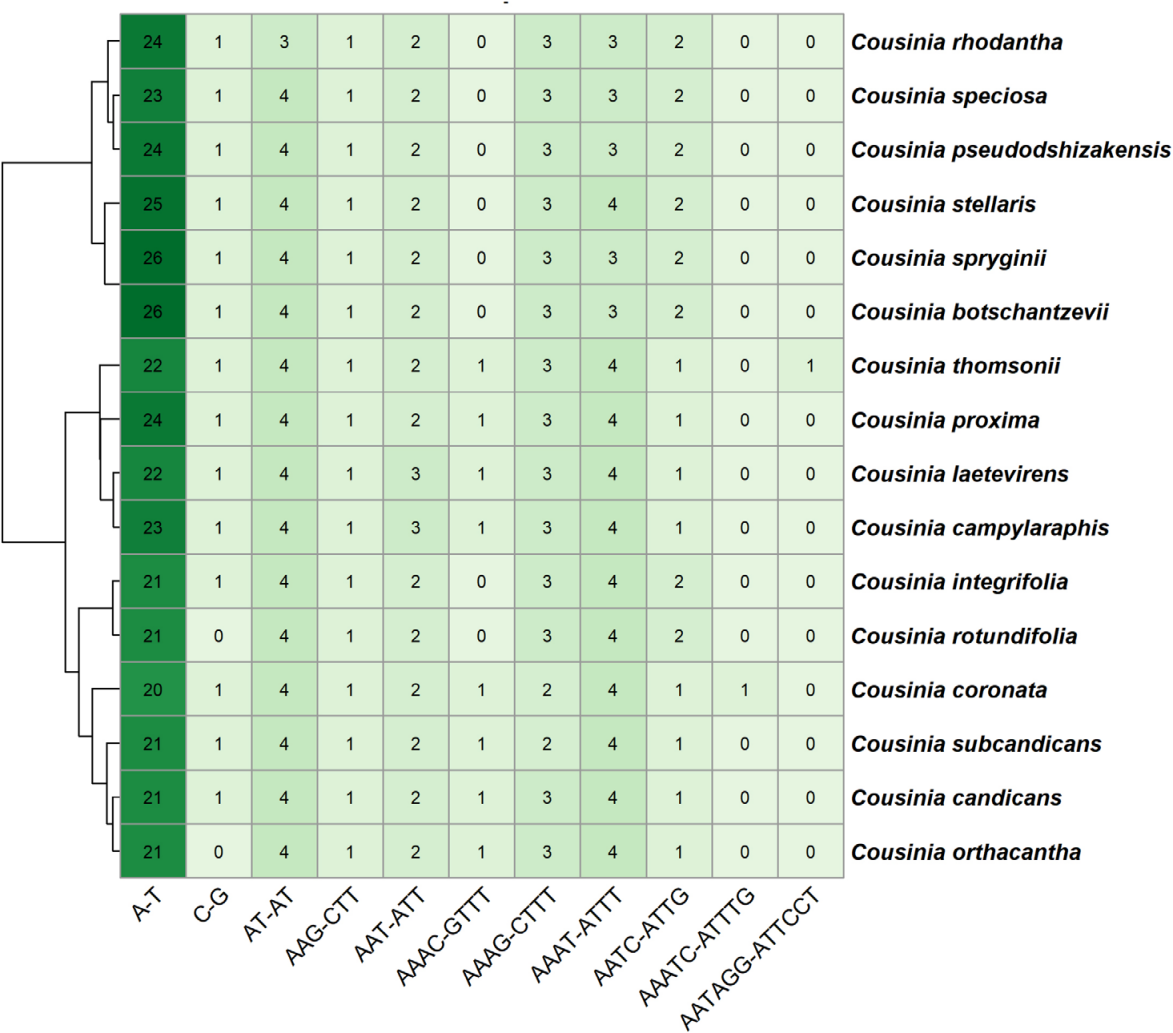
Heatmap of simple sequence repeat (SSR) motif abundance in *Cousinia* chloroplast genomes with hierarchical clustering.

Analysis of cp genomes revealed a total of 11 different SSR (simple sequence repeat) motifs, including 2 mononucleotide, 1 dinucleotide, 2 trinucleotide, 4 tetranucleotide, 1 pentanucleotide, and 1 hexanucleotide motifs (Figure 5 and File S2). The most frequent motif type was the A/T mononucleotide repeat, which occurred with high frequency across all species, ranging from 20 repeats in *C. coronata* to 26 in *C. spryginii* and *C. botschantzevii*. In contrast, C/G‐type mononucleotide SSRs were relatively rare, occurring only once in all examined species, and were completely absent in *C. rotundifolia* and *C. orthacantha*. The pentanucleotide motif AAATC–ATTTG was found exclusively in *C. coronata* in *trnE‐UUC—rpoB*, while the hexanucleotide motif AATAGG–ATTCCT was unique to *C. thomsonii* in *rpoC2*. Cluster analysis based on simple sequence repeat (SSR) profiles revealed distinct groupings among the *Cousinia* species. Notably, *C. spryginii* and *C. botschantzevii* exhibited nearly identical SSR profiles, while *C. stellaris* displayed a highly similar profile, suggesting close genetic affinity among these taxa. Species from the sections *Alpinae* and *Tianschanicae*— *C. rhodantha* , *C. speciosa* , and *C. pseudodshizakensis*—formed a strongly supported cluster, differing only at a single mono‐ and dinucleotide locus. Likewise, *C. laetevirens* and *C. campylaraphis* shared fully concordant SSR profiles across all mono‐, di‐, tri‐, and tetranucleotide loci, with the sole distinction being an additional A–T repeat in *C. campylaraphis*. Similarly, *C. integrifolia* and *C. rotundifolia* exhibited highly comparable SSR patterns, differing primarily in the C–G motif, which was absent in *C. rotundifolia* .

### 3.6 Phylogenetic Analysis

**FIGURE 6:**
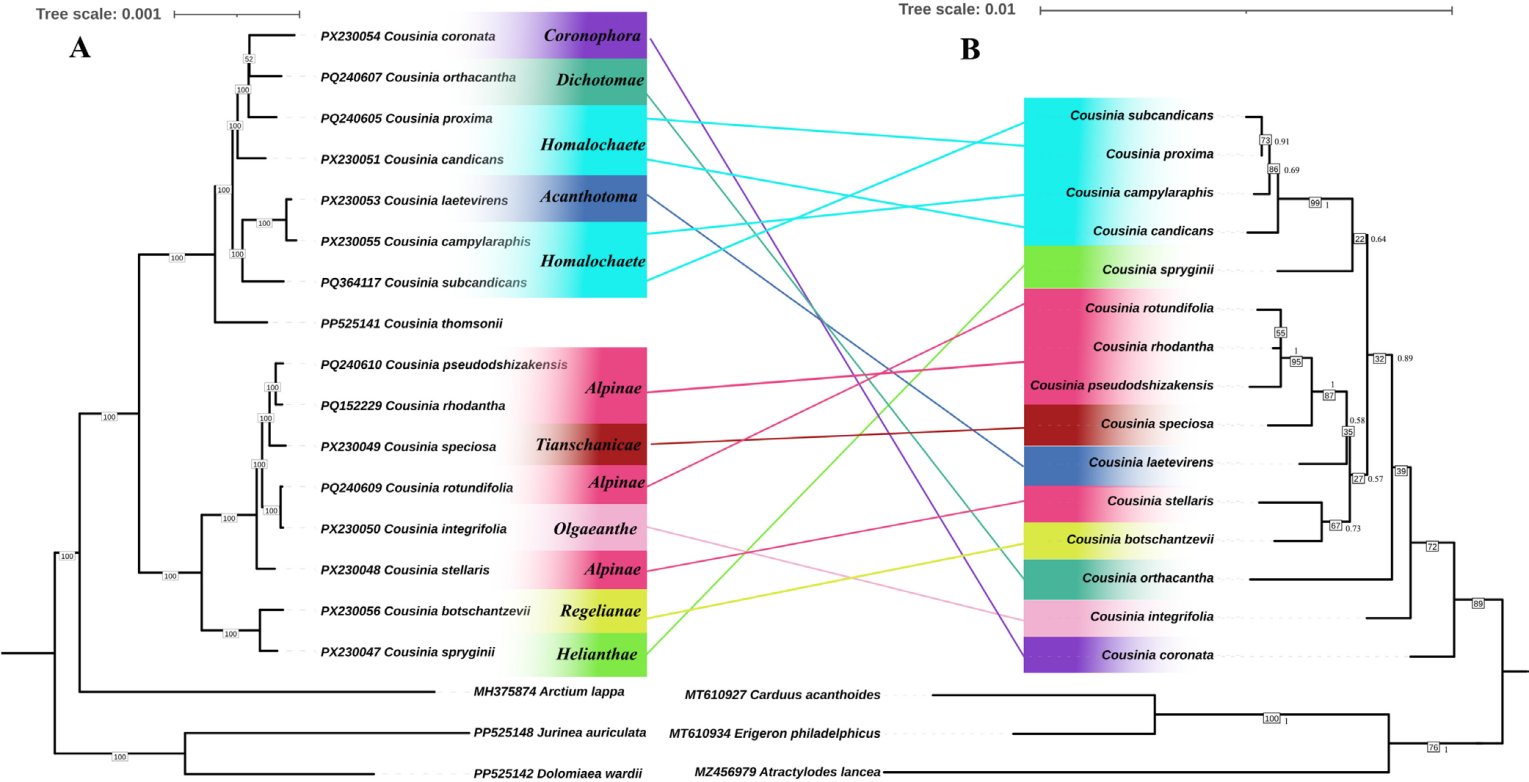
Comparison of phylogenetic trees of *Cousinia* species inferred from complete chloroplast genomes (A) and nuclear ribosomal nrDNA sequences (B).

Analyses based on the cp genome indicated no support for the monophyly of the morphologically defined sections within *Cousinia* (Figure 6A). Species attributed to section *Homalochaete* did not form an exclusive group; instead, four of them were recovered in a well‐supported clade (BS = 100) together with taxa representing sections *Acanthotoma*, *Dichotomae*, and *Coronophora*. All members of this clade are confined to the Pamir–Alay region. The closest relative of this assemblage was *Cousinia thomsonii*, a species whose native range extends from Afghanistan eastward to Nepal and southern Tibet. A similar pattern emerged for section *Alpinae*. Four species traditionally placed in this section grouped with representatives of sections *Tianschanicae* and *Olgaeanthe*, forming a second, distinct clade composed entirely of Pamir–Alay endemics. This clade was inferred to be sister to a lineage comprising *Cousinia botschantzevii* (section *Regelianae*) and *Cousinia spryginii* (section *Heliantheae*), both of which likewise exhibit strict endemism to the Pamir–Alay region.

In contrast, the nrDNA‐based phylogeny better supported the monophyly of the morphologically defined sections of *Cousinia*, although most nodes showed lower bootstrap values compared with the chloroplast tree (Figure 6B). The four species assigned to section *Homalochaete* formed a coherent and well‐defined clade. The single representatives included from sections *Acanthotoma*, *Dichotomae*, *Coronophora*, *Olgaeanthe*, and *Heliantheae* each occupied isolated lineages without intermixing with other sections, fully consistent with their morphological delimitation. Only two exceptions were observed: *C. speciosa* (section Tianschanicae) nested within the Alpinae clade (ML = 87), and *C. stellaris* (section Alpinae) clustered with *C. botschantzevii* of section *Regelianae*, forming a moderately supported monophyletic lineage (ML = 67).

### 3.7 The Form of the Anther Appendages

**FIGURE 7:**
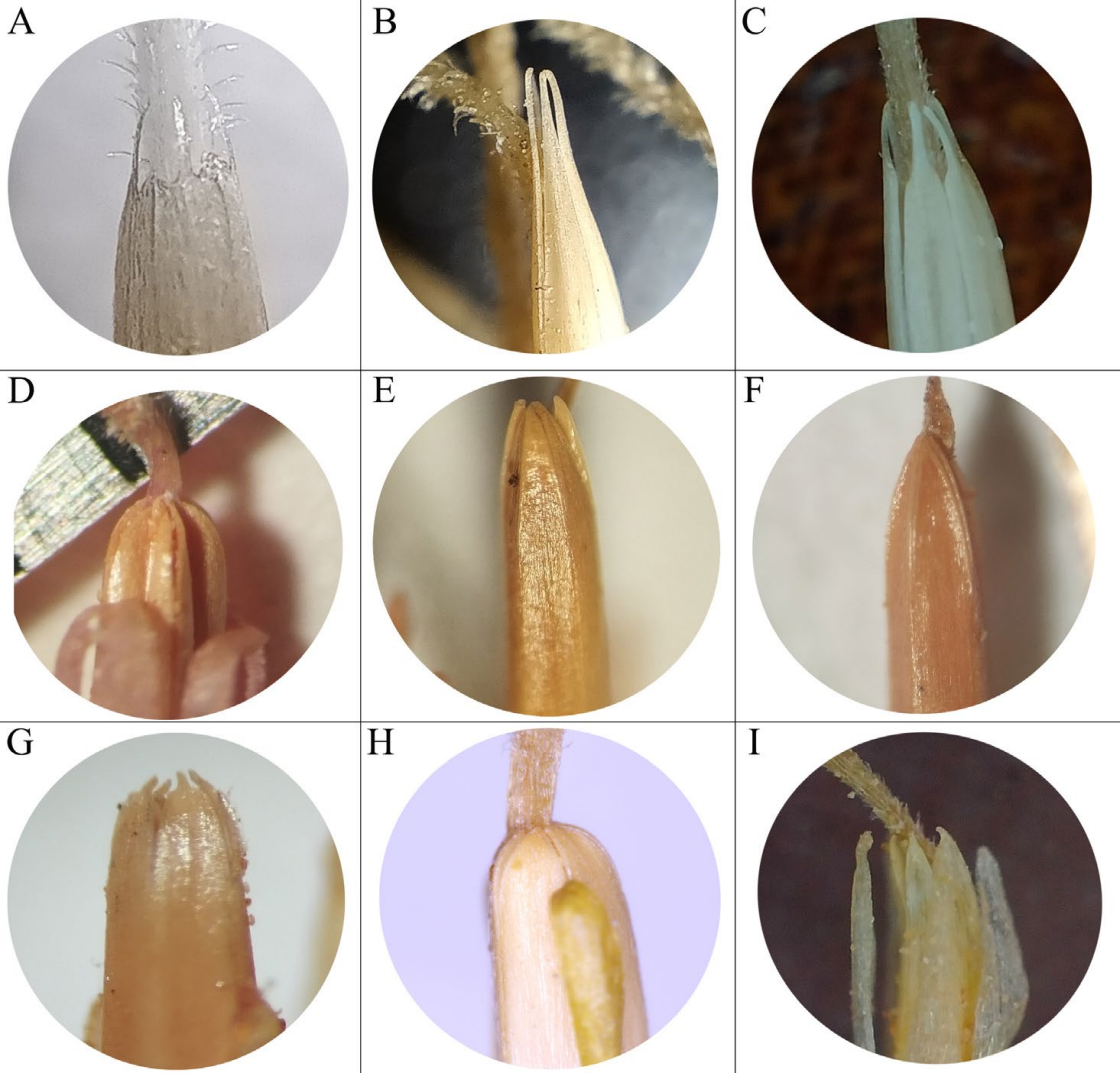
Variation in anther appendage morphology among *Cousinia* species. (A) Irregularly dentate appendages, (B) Narrowly elongate, smooth appendages, (C) Narrowly elongate appendages with a toothed apex, (D) Short, smoothly rounded lobes forming a dome‐shaped or arched apex, (E) Narrowly elongate, not arched in outline, (F) Narrowly elongate, arched in outline, (G) Crown‐shaped apex with a toothed apex, *H. apex* short, entire, and rounded, (I) Elongate appendages with a very small or absent apical tooth.

We examined the anther–appendage morphology of more than 40 species of *Cousinia* (File S3) and distinguished nine discrete structural groups (Figure 7). Group A, consisting of irregularly dentate appendages, was recorded only in *C. botschantzevii* Juz. ex Tscherneva and *C. regelii* C. Winkl. of section *Regelianae*. Group B, characterized by narrowly elongate and smooth appendages, comprised five species: *C. spryginii* Kult. (section *Helianthae*), *C. ferghanensis* Bornm. and *C. simulatrix* C. Winkl. (section *Subappendiculatae*), *C. divaricata* C. Winkl. (section *Leiacanthos*), and *C. princeps* Franch. (section *Alpinae*). Group C, defined by narrowly elongate appendages with a toothed apex, included 12 species— *C. proxima* , *C. corymbosa* , *C. podophylla* , *C. coerulea* , *C. campylaraphis*, *C. subcandicans*, and *C. litwinowiana* from section *Homalochaete*; *C. verticillaris* , *C. laetevirens*, *C. sarawschanica*, and *C. splendida* from section *Acanthotoma*; and *C. magnifica* from section *Racemosae*. Group D, possessing short, smoothly rounded lobes forming a dome‐shaped or arched apex, was restricted to four species of section *Alpinae*: *Cousinia stellaris* Bornm., *C. pseudodshizakensis* Tschern. & Vved., *C. rotundifolia* C. Winkl., and *C. grigoriewii*. Group E, consisting of narrowly elongate appendages without an arched outline, was observed in *C. rosea* Kult., *C. alpina* Bunge, and *C. calva* Juz. (section *Alpinae*), *C. speciosa* C. Winkl. (section *Tianschanicae*), and *C. dubia* Popov and *C. submutica* Franch. (section *Jurineopsis*). Group F, defined by long, acute appendages with an arched outline, included *C. integrifolia* Franch. (section *Olgaeanthe*), *C. coronata* Franch. and *C. radians* Bunge (section *Coronophora*), and *C. outichaschensis* Franch., *C. buphthalmoides* Regel (together with its synonym *C. auriculata* Hook.f.), and *C. rava* C. Winkl. of section *Alpinae*. Group G, a crown‐shaped apex bearing teeth, was restricted to *C. psammophila* Kult. of section *Chrysoptera*. Group H, having a short, entire, and rounded apex, was observed in *C. pseudolanata* Popov ex Tscherneva and *C. lanata* C. Winkl. of section *Racemosae*. Group I, defined by elongate appendages with a minute or absent apical tooth, characterized four species of section *Dichotomae*: *C. tedshenica* Tscherneva, *C. sylvicola* Bunge, *C. patentispina* Tscherneva, and *C. orthacantha* Tscherneva.

## 4 Discussion

The present study represents cp genome analysis of *Cousinia* to date, expanding our understanding of cp genome structural variation, codon usage evolution, and phylogenetic relationships in this taxonomically challenging genus.

The structural architecture of *Cousinia* cp genomes, including their quadripartite configuration, approximate length (~152 kb), and overall GC proportion (37%), aligns closely with patterns widely documented within Asteraceae (Mahai et al. 2024; Nyamgerel et al. 2024; Xing et al. 2025; Zhong et al. 2023). Both gene complement and intron distribution exhibit a high degree of uniformity across examined taxa, indicating strong structural conservation. Such stability is consistent with the evolutionary persistence typically reported for Cardueae (Abdullah et al. 2026) and angiosperm cp genomes (Jansen and Ruhlman 2012), suggesting limited large‐scale genomic restructuring within the genus.

Assessment of synonymous codon usage across the cp genomes of 16 *Cousinia* species demonstrated a strongly conserved pattern characterized by a pronounced preference for A/T‐terminating codons. Such compositional asymmetry is a common feature of Asteraceae cp genomes and corresponds to their overall AT‐rich nucleotide composition (Nyamgerel et al. 2024; Abdullah et al. 2025, 2026). Codons ending in G or C were comparatively infrequent, indicating that base composition exerts a substantial influence on codon selection.

Analysis of nucleotide diversity revealed that *Cousinia* cp genomes are highly conserved, with Pi values ranging from 0 to 0.00918. These estimates fall within the range reported for other Cardueae genera, including *Saussurea* (He et al. 2023), indicating comparable levels of cp genome stability within thistle‐like lineages. Regions exhibiting elevated variability—*trnK‐UUU–rps16*, *trnE‐UUC–rpoB*, *trnS‐GGA–rps4*, *trnL‐UAA–trnF‐GAA–ndhJ*, *rbcL*, *ndhF*, *rpl32–trnL‐UAG*, *ycf1*, *and rbcL*—coincide with loci frequently identified as rapidly evolving in Asteraceae (Shen et al. 2020; Kim et al. 2024). The recurrence of these divergence hotspots across related genera highlights their potential utility as informative markers for species discrimination and phylogenetic inference. Accordingly, these regions represent promising candidates for future chloroplast‐based barcoding initiatives and for reconstructing the historical biogeography of *Cousinia*.

Microsatellite screening of the cp genomes identified a total of 639 SSR loci across 16 *Cousinia* species, comprising 11 distinct motif categories. Mononucleotide repeats composed of A/T bases represented the dominant class, whereas C/G‐rich motifs occurred infrequently and were completely absent in several taxa. Such enrichment of A/T repeats is a well‐documented characteristic of angiosperm cp genomes and has also been reported in other genera of Asteraceae, including *Jacobaea* (Doorduin et al. 2011), *Artemisia* (Shen et al. 2017), *Dendrosenecio* (Gichira et al. 2019), *Saussurea* (Zhang et al. 2019; Nyamgerel et al. 2024), and *Dolomiaea* (Shen et al. 2020). SSR‐based clustering revealed several species groupings that partially align with chloroplast‐based phylogeny. However, these patterns should be interpreted with caution. Due to the high mutation rate and potential homoplasy of SSR loci, such clustering does not necessarily reflect true phylogenetic relationships. These markers can aid in a better understanding of the genetic diversity.

Phylogenetic reconstruction based on complete cp genomes did not recover all morphologically circumscribed *Cousinia* sections as monophyletic, indicating discordance between chloroplast‐derived topologies and traditional sectional classifications. Such incongruence may reflect processes including chloroplast capture, historical introgression, or incomplete lineage sorting. Given the predominantly maternal inheritance of cp DNA, chloroplast‐based phylogenies may capture only a partial representation of species evolutionary history, particularly in groups where hybridization and reticulate evolution are prevalent (Rieseberg and Soltis 1991; Soltis and Kuzoff 1995; Soltis and Soltis 1998; Tsitrone et al. 2003). Evidence of interspecific hybridization within *Cousinia*, occurring both within and between major clades, further supports this interpretation. Notably, approximately 7%–10% of *Cousinia* species have been reported to participate in interspecific hybridization (Mehregan and Kadereit 2009). Moderate levels of hybridization may contribute to chloroplast–nuclear discordance and complicate phylogenetic inference within the genus. In contrast, the nuclear nrDNA dataset yielded a topology more consistent with morphology‐based sectional delimitations, although nodal support values were generally lower. An exception was *Cousinia stellaris* (section *Alpinae*), which grouped with representatives of section *Regelianae* in both chloroplast and nuclear reconstructions. This consistent placement across datasets suggests that *C. stellaris* may be misassigned within the current sectional framework. The closer correspondence between nrDNA phylogeny and morphological classification likely reflects the biparental inheritance of nuclear markers, which can provide a more comprehensive representation of organismal evolutionary history (Álvarez and Wendel 2003; Feliner and Rosselló 2007). Together, these findings underscore the importance of integrating chloroplast and nuclear evidence when reconstructing phylogenetic relationships in taxonomically complex plant lineages.

The observed diversity of anther appendage morphology within *Cousinia* demonstrates pronounced structural differentiation among sections. Certain appendage types were confined to particular lineages (*Regelianae*), whereas others were distributed across unrelated sections. In Flora of the USSR (Tscherneva 1962), *C. pseudolanata*, *C. lanata* , and *C. magnifica* were originally assigned to section *Acanthotoma* (formerly *Alpinae*). Later, in Conspectus florae Asiae Mediae (Tscherneva 1993), these three species were transferred to the newly established section *Racemosae*. The transfer of *C*. *pseudolanata* and *C. lanata* is supported by their similar anther appendage morphology, characterized by short, entire, and rounded apices. However, *C. magnifica* possesses narrowly elongate appendages with a toothed apex, aligning morphologically with species of *Acanthotom*a. Consequently, we recommend that *C. magnifica* be retained within section *Acanthotoma*. The morphological heterogeneity of section *Alpinae* is evident from the diversity of anther appendage forms represented among its species. Members of this section occur in several morphological groups: *C. princeps* in Group B (narrowly elongate, smooth appendages); *C. stellaris* , *C. pseudodshizakensis*, *C. rotundifolia* , and *C. grigoriewii* in Group D (short, dome‐shaped, arched in outline); *C. rosea, C. alpina*, and *C. calva* in Group E (narrowly elongate, not arched in outline); and *C. outichaschensis, C. buphthalmoides*, and *C. rava* in Group F (long, arched appendages with acute apices). These findings suggest that the current circumscription of *Alpinae* may not accurately reflect evolutionary relationships and that both morphological and molecular characters should be considered in future taxonomic revisions of *Cousinia*.

## 5 Conclusion

This study provides an integrative assessment of *Cousinia* in the Pamir–Alay region using cp genome, nrDNA, and morphological data. Although cp genomes are structurally conserved, phylogenetic analyses reveal clear incongruence between chloroplast and nuclear signals. Importantly, section *Homalochaete* is recovered as monophyletic in the nrDNA phylogeny, forming a well‐supported and morphologically coherent lineage. This supports its recognition as a natural taxonomic unit when nuclear evidence is considered, despite its nonmonophyly in cp genome‐based analyses. In contrast, sections such as *Alpinae* remain highly heterogeneous and require subdivision into smaller, more natural groups based on combined chloroplast, nuclear, and morphological evidence. Morphological data further support transferring *Cousinia magnifica* back to *Acanthotoma* and confirm the coherence of *Racemosae*. Highly variable chloroplast regions identified in this study (*trnF‐GAA*, *trnP‐UGG*, *ycf1*, *atpH*, *rbcL*, *rnL‐UAG–ccsA*, *rps11–rpl36*) are recommended as candidate DNA barcodes for *Cousinia*. Future studies should substantially expand taxonomic sampling. At present, only a limited number of cp genomes (16) are available, representing a very small fraction of the more than 600 species recognized in *Cousinia*. Broader sampling across sections and geographic regions, combined with nuclear genomic data (e.g., low‐copy genes, Hyb‐Seq), will be essential to resolve phylogenetic relationships more robustly. Overall, this work provides a fundamental framework for future large‐scale phylogenetic and taxonomic revision of this complex genus.

## Author Contributions

**Bobur Karimov:** conceptualization (equal), data curation (lead), formal analysis (lead), funding acquisition (lead), methodology (equal), visualization (equal), writing – original draft (lead), writing – review and editing (equal). **Diyorjon Hamrayev:** conceptualization (equal), data curation (equal), formal analysis (equal), methodology (equal), visualization (equal), writing – review and editing (equal). **Husniddin Esanov:** data curation (equal), investigation (lead), methodology (supporting). **Alijon Eshonkulov:** formal analysis (equal), visualization (lead), writing – review and editing (supporting). **Oybek Omonov:** methodology (equal), validation (equal), writing – review and editing (supporting). **Nodira Boboyeva:** data curation (supporting), methodology (supporting), writing – review and editing (supporting). **Damira Karimova:** methodology (supporting), validation (supporting), writing – review and editing (supporting). **Abdullajon Umedov:** data curation (supporting), investigation (equal), methodology (supporting), writing – original draft (equal). **Temur Asatulloev:** formal analysis (supporting), software (lead), validation (equal). **Ziyoviddin Yusupov:** conceptualization (lead), project administration (equal), supervision (lead), writing – review and editing (equal). **Komiljon Sh. Tojibaev:** project administration (lead), supervision (lead), writing – review and editing (equal).

## Funding

The authors have nothing to report.

## Conflicts of Interest

The authors declare no conflicts of interest.

## Supporting information


**Figure S1:** Patterns of nucleotide diversity (π) across coding (A) and noncoding (B) regions of *Cousinia* cp genomes.

**File S1:** Comprehensive analysis of codon usage patterns in the chloroplast genomes of *Cousinia* species.

**File S2:** Genome‐wide characterization and distribution of simple sequence repeats (SSRs) in *Cousinia* cp genomes.

**File S3:** Comparative morphological analysis of anther appendage shape variation in *Cousinia* species.

## Data Availability

The raw sequencing data generated in this study have been deposited in the NCBI Sequence Read Archive (SRA) under BioProject accession number PRJNA1124612 (*Cousinia* genome sequencing and assembly, Tree of Life Uzbekistan; multispecies). All other data generated or analyzed during this study are included in this published article and its Supporting Information.
