# Supplementary figures and images for: Comparative Chloroplast Genomics of Cousinia (Asteraceae) With Nine Newly Sequenced Endemic Species From Central Asia
Source: Ecol Evol. 2026 Apr 29;16(5):e73589. doi: 10.1002/ece3.73589 (PMC13127105; doi:10.1002/ece3.73589)

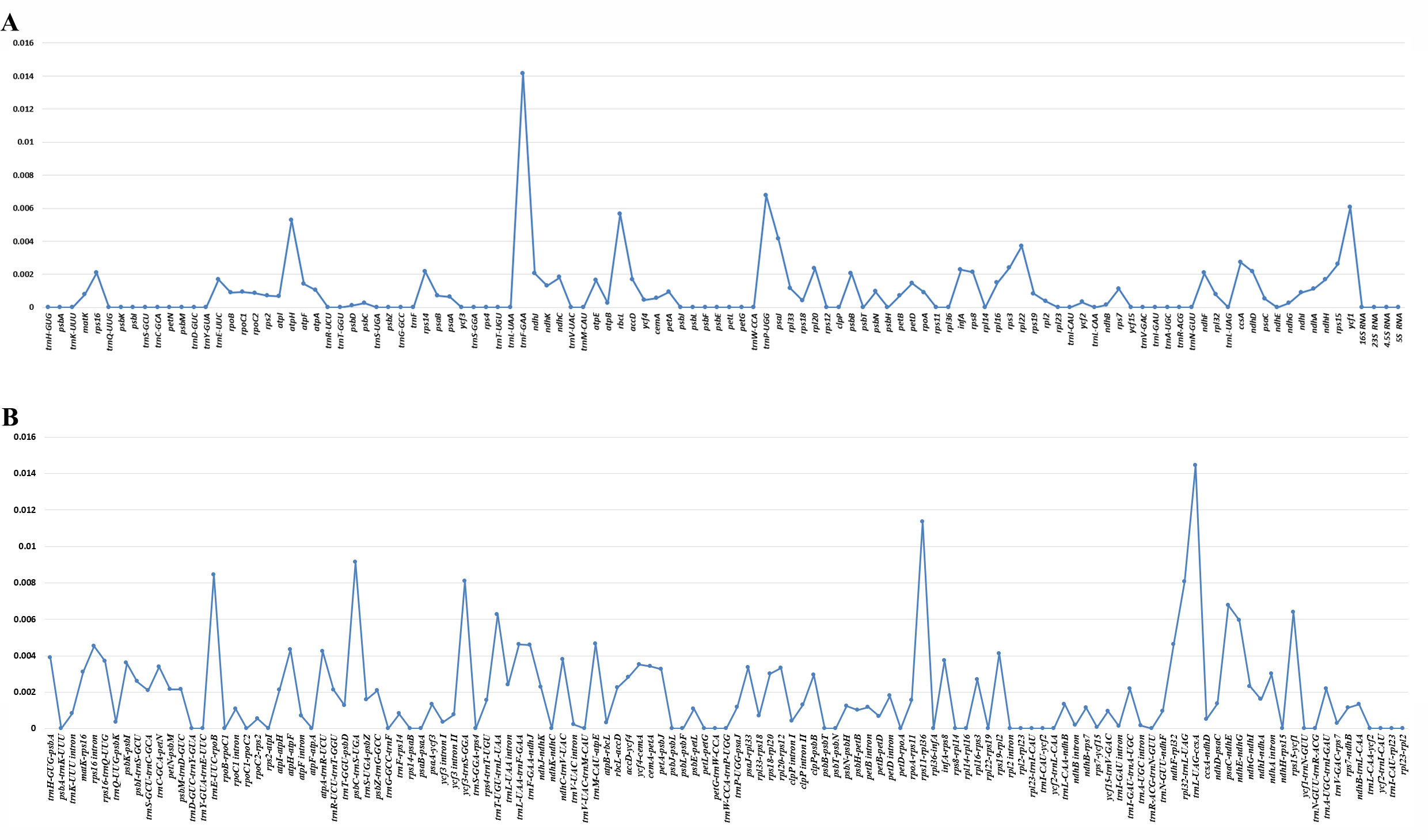

Supplement: Supplementary file 1 — Figure S1: Patterns of nucleotide diversity (π) across coding (A) and noncoding (B) regions of Cousinia cp genomes. [file ECE3-16-e73589-s004.jpg]
